# HIV Care Visits and Time to Viral Suppression, 19 U.S. Jurisdictions, and Implications for Treatment, Prevention and the National HIV/AIDS Strategy

**DOI:** 10.1371/journal.pone.0084318

**Published:** 2013-12-31

**Authors:** H. Irene Hall, Tian Tang, Andrew O. Westfall, Michael J. Mugavero

**Affiliations:** 1 Division of HIV/AIDS Prevention, National Center for HIV/AIDS, Viral Hepatitis, STD and TB Prevention, Centers for Disease Control and Prevention, Atlanta, Georgia, United States of America; 2 ICF International, Atlanta, Georgia, United States of America; 3 Center for AIDS Research, University of Alabama at Birmingham, Birmingham, Alabama, United States of America; 4 Division of Infectious Diseases, Department of Medicine, University of Alabama at Birmingham, Birmingham, Alabama, United States of America; The University of Hong Kong, Hong Kong

## Abstract

**Objective:**

Early and regular care and treatment for human immunodeficiency virus (HIV) infection are associated with viral suppression, reductions in transmission risk and improved health outcomes for persons with HIV. We determined, on a population level, the association of care visits with time from HIV diagnosis to viral suppression.

**Methods:**

Using data from 19 areas reporting HIV-related tests to national HIV surveillance, we determined time from diagnosis to viral suppression among 17,028 persons diagnosed with HIV during 2009, followed through December 2011, using data reported through December 2012. Using Cox proportional hazards models, we assessed factors associated with viral suppression, including linkage to care within 3 months of diagnosis, a goal set forth by the National HIV/AIDS Strategy, and number of HIV care visits as determined by CD4 and viral load test results, while controlling for demographic, clinical, and risk characteristics.

**Results:**

Of 17,028 persons diagnosed with HIV during 2009 in the 19 areas, 76.6% were linked to care within 3 months of diagnosis and 57.0% had a suppressed viral load during the observation period. Median time from diagnosis to viral suppression was 19 months overall, and 8 months among persons with an initial CD4 count ≤350 cells/µL. During the first 12 months after diagnosis, persons linked to care within 3 months experienced shorter times to viral suppression (higher rate of viral suppression per unit time, hazard ratio [HR] = 4.84 versus not linked within 3 months; 95% confidence interval [CI] 4.27, 5.48). Persons with a higher number of time-updated care visits also experienced a shorter time to viral suppression (HR = 1.51 per additional visit, 95% CI 1.49, 1.52).

**Conclusions:**

Timely linkage to care and greater frequency of care visits were associated with faster time to viral suppression with implications for individual health outcomes and for secondary prevention.

## Introduction

Suppression of human immunodeficiency virus (HIV) plasma viral load reduces HIV-related morbidity and mortality and can prevent onward transmission of HIV [1]. However, less than 30% of persons living with HIV in the United States have a suppressed viral load [2], [3]. Viral suppression is the outcome of sequential steps in the continuum of care, including diagnosis, linkage and retention in HIV care, and antiretroviral treatment (ART) prescription and adherence [2]–[4].

Of the more than 1.1 million people living with HIV in the United States, an estimated 200,000 are not aware of their infection and therefore do not receive the benefits of care and treatment [5]. However, even among those diagnosed, an estimated 1 in 5 are not promptly linked to care and less than half are in regular HIV care [6], [7]. Studies have shown that persons who enter care soon after diagnosis and maintain regular care initiate ART earlier, have better adherence to ART, and have better health outcomes [8]–[11]. In addition, adherence to clinic visits was associated with earlier viral suppression and lower cumulative viral load burden [10]. However, these findings were based on persons who had already been linked to HIV medical care at selected clinics, and information is not available at the population level on the association between early entry to HIV care after diagnosis or frequency of HIV care visits and the time to viral suppression. Shortening the time from HIV diagnosis to care entry and viral suppression has important implications for HIV treatment and prevention as this would shorten the time individuals could potentially transmit the virus. However, limited evidence has demonstrated a benefit to health outcomes among persons linked to care within 3 months of HIV diagnosis, a goal set forth by the National HIV/AIDS Strategy and a quality indicator in a recent Institute of Medicine Report [12], [13].

Population-based HIV surveillance data can be used to determine care visits among persons with HIV based on reports of CD4 and viral load test results, and test results can be used to determine viral load and time to viral suppression. We used data from the National HIV Surveillance System to determine the associations between entry to care and number of care visits with time to viral suppression. We hypothesized that linkage to care within 3 months of HIV diagnosis and greater frequency of care visits would be significantly associated with faster time to viral suppression.

## Methods

HIV infection is reportable in all 50 states, the District of Columbia, and six U.S. dependent areas. All cases are reported from state and local health departments to the Centers for Disease Control and Prevention (CDC) without identifying information. Assessments and elimination of duplicate reports occur both on the state and national level. However, not all areas have mandatory reporting of all HIV-related laboratory tests, including all values of CD4 cell counts or percentages and viral load tests. Using data from 19 jurisdictions (California [Los Angeles County and San Francisco only], Delaware, District of Columbia, Georgia, Hawaii, Illinois, Indiana, Iowa, Louisiana, Michigan, Minnesota, Missouri, Nebraska, New Hampshire, New York, North Dakota, South Carolina, West Virginia, Wyoming) with mandatory laboratory reporting of HIV-related tests and reporting of all tests to national HIV surveillance, we assessed factors associated with viral suppression in persons >12 years old who were diagnosed with HIV during 2009. Persons were followed through December 2011 and data were reported to CDC through December 2012.

We assessed time to viral suppression (defined as ≤200 copies/mL) after HIV diagnosis, with an observation period from diagnosis through December 2011. Patients were followed until the earliest of viral suppression, death or the administrative censoring date of December 2011. Patients who died or did not attain viral suppression before December 2011 were censored. Using data on CD4 and viral load test results, we defined two care indicators: 1) early linkage to care (≥1 CD4 or viral load test result within 3 months of diagnosis), a goal of the National HIV/AIDS Strategy and a quality indicator defined by the Institute of Medicine [12], [13], and 2) the number of care visits within the observation period (number of CD4 or viral load test results) but before or at viral suppression. Two or more CD4 or viral load test results collected in the same calendar month were considered as one care visit. We assessed outcomes overall and adjusted for sex, race/ethnicity (black or African American [hereafter referred to as ‘black’], Hispanic or Latino, white, multiple/other race), age, transmission category (male-to-male sexual contact, injection drug use, male-to-male sexual contact and injection drug use, heterosexual contact, and other), and severity of disease at diagnosis (CD4 count <200 cells/µL or opportunistic illness; CD4 count 200–350 cells/µL; CD4 count >350 cells/µL; and unknown, based on reported CD4 test results or opportunistic illnesses within 3 months of diagnosis [for 0.7% of persons only CD4 percentage was available and we assigned count equivalents as follows: CD4 percent <14% as CD4 count of <200 cells/µL, CD4 percent 14%–24% as CD4 count of 200–350 cells/µL, and CD4 percent >24% as CD4 count of >350 cells/µL]) [14], [15].

### Statistical Analysis

Kaplan-Meier analyses were used to generate survival curves and to estimate the median time to viral suppression overall and by entry to care within 3 months and CD4 count at baseline. We used Cox proportional hazards models to assess factors associated with time to first suppressed viral load. Because entry to care and subsequent care visits are on the causal pathway from HIV diagnosis to viral suppression, two separate Cox models were constructed with one model evaluating care entry within 3 months and the other the time-updated cumulative number of care visits. Adjusted models controlled for covariates listed above. The proportionality of hazard assumption was tested for all covariates included in the Cox models. Since there was a significant interaction between linkage to care within 3 months of diagnosis and time to viral suppression (i.e., non-proportional hazards), we added a time interaction term and assessed the effect of linkage to care on two time intervals (≤12 months, and >12 months). For the entry to care variable the proportional hazards assumption was met for the >12 months time period. However, the proportional hazards assumption was not met for the ≤12 months time period. After examination of the plot of the kernel-smoothed hazard functions it was clear that although not proportional, the hazard rate for those who entered care within 3 months was consistently higher than the rate for those that did not enter care within 3 months and thus it would be reasonable to report what is essentially the average hazard ratio over this time period. Because they accumulate over time, the number of care visits was handled as a time-updated covariate in the models. Because the prevailing treatment guidelines during the study period (2009) recommended ART initiation at a CD4 count ≤350 cells/µL, we conducted sensitivity analyses limited to participants with an initial CD4 count below this threshold. All analyses were performed using SAS version 9.3 (SAS Institute Inc., Cary, NC), with statistical significance defined as a 2-sided p-value<0.05.

Of the persons >12 years old and diagnosed in 2009 in the 19 jurisdictions, 141 (0.82%) were excluded from the analysis because the date of diagnosis was incomplete (missing month of diagnosis). For the 17,028 persons diagnosed with HIV included in the analyses, information for transmission category was not available for 5,648; these were retained in the analyses in the “Other” category. About 0.4% (1,175 of a total of 277,580 CD4 or viral load tests) of CD4 or viral load tests were excluded because their month difference could not be determined between the diagnosis date and the specimen collection date (month of specimen collection date is missing), and 0.04% (38 of a total of 103,361 viral load test results) of viral load test results were not valid and were not included when calculating time to viral suppression.

## Results

Of 17,028 persons diagnosed with HIV during the calendar year 2009 and followed through December 2011 in the 19 jurisdictions, 51.4% were black, 19.0% were Hispanic or Latino, 24.8% were white and 4.7% were of other races (Table 1). The majority were male (77.5%) and 47.3% had infection attributed to male-to-male sexual contact. Nearly a third (29.4%) had a baseline CD4 count of <200 cells/µL or an AIDS diagnosis based on opportunistic illness.

**Table 1:** Characteristics of adolescents and adults^a^ diagnosed with HIV infection, 2009, 19 U.S. jurisdictions.

	Total^b^	Had a VL test^c^		Had a suppressed VL test^d^		
Characteristic	No. (%)	No.	% from theoverallpopulation	No.	% from the overall population	% from the persons with a VL test
**Sex**						
Male	13,201 (77.5%)	11,266	85.3	7,495	56.8	66.5
Female	3,827 (22.5%)	3,278	85.7	2,209	57.7	67.4
**Age at diagnosis (yrs)**	36.4±12.3					
13–24	3,484 (20.5%)	2,973	85.3	1,700	48.8	57.2
25–34	4,697 (27.6%)	4,042	86.1	2,671	56.9	66.1
35–44	4,325 (25.4%)	3,697	85.5	2,627	60.7	71.1
45–54	3,161 (18.6%)	2,692	85.2	1,908	60.4	70.9
55+	1,361 (8.0%)	1,140	83.8	798	58.6	70
**Race/ethnicity**						
Black/African American	8,757 (51.4%)	7,254	82.8	4,469	51	61.6
Hispanic/Latino	3,236 (19.0%)	2,748	84.9	1,937	59.9	70.5
White	4,228 (24.8%)	3,832	90.6	2,792	66	72.9
Other	807 (4.7%)	710	88	506	62.7	71.3
**Transmission category**						
Male-to-male sexual contact	8,062 (47.3%)	7,129	88.4	4,839	60	67.9
Injection drug use	751 (4.4%)	634	84.4	403	53.7	63.6
Male-to-male sexual contact andinjection drug use	392 (2.3%)	356	90.8	231	58.9	64.9
Heterosexual contact	2,174 (12.8%)	1,959	90.1	1,322	60.8	67.5
Other^e^	5,649 (33.2%)	4,466	79.1	2,909	51.5	65.1
**Baseline CD4 count** f						
<200 cells/µL or Stage 3, AIDS	5,009 (29.4%)	4,663	93.1	3,524	70.4	75.6
200–350 cells/µL	2,052 (12.1%)	1,986	96.8	1,520	74.1	76.5
>350 cells/µL	5,118 (30.1%)	4,949	96.7	2,949	57.6	59.6
Unknown	4,849 (28.5%)	2,946	60.8	1,711	35.3	58.1
**Care entry within 3 months**						
**of diagnosis**						
No	3,984 (23.4%)	2,039	51.2	1,176	29.5	57.7
Yes	13,044 (76.6%)	12,505	95.9	8,528	65.4	68.2
**Total**	**17,028 (100%)**	**14,544**	**85.4**	**9,704**	**57**	**66.7**

^a^ 17028 adolescents and adults (aged >12 years) diagnosed in 2009, with followed up through December 31, 2011.

^b^ Percentage reflects the column percentage; numerator is the number of persons in the group and denominator is the total number of persons diagnosed with HIV in 2009. Mean (standard deviation) was also calculated for age at HIV diagnosis.

^c^ Had a viral load (VL) test within follow-up period. Percentage reflects number of persons with a VL test among the total population.

^d^ Had a suppressed VL within follow-up period. For percentage among total population, the denominator is the total population (number of persons diagnosed with HIV in that group). For the percentage among persons with VL test, the denominator is the number of persons with a VL test in that group.

^e^ Includes 5,648 persons with unknown transmission category.

^f^ CD4 test result or opportunistic illnesses within 3 months of diagnosis; persons without a CD4 test result or OI within 3 months of diagnosis are classified as “unknown”.

About three quarters of persons diagnosed with HIV (76.6%) were linked to care within 3 months of diagnosis. Of the total of 17,028 persons, 1,828 had no evidence of care during the observation period. For persons who entered care within the observation period, the mean time from diagnosis to care entry was 6 months (Kaplan-Meier estimate), and one month for those who entered care within 3 months and 22 months for those who entered care later.

**Figure 1:**
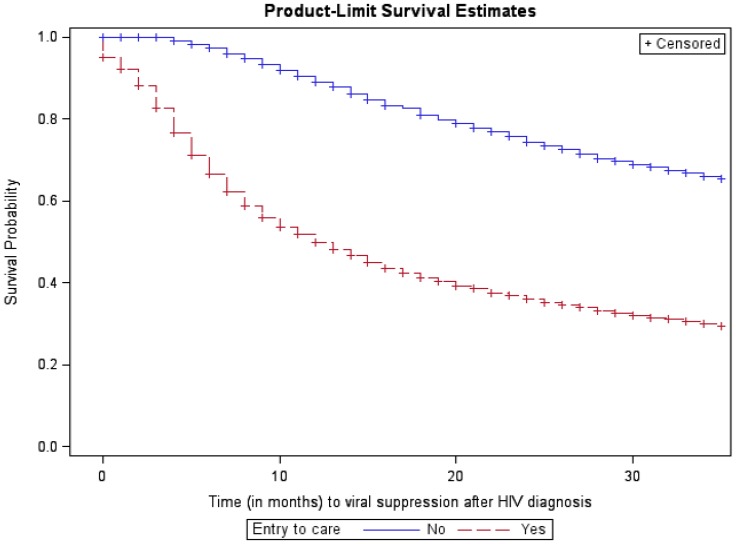
Kaplan-Meier survival curve for time from HIV diagnosis to viral suppression, by entry to care within 3 months of HIV diagnosis.

**Figure 2:**
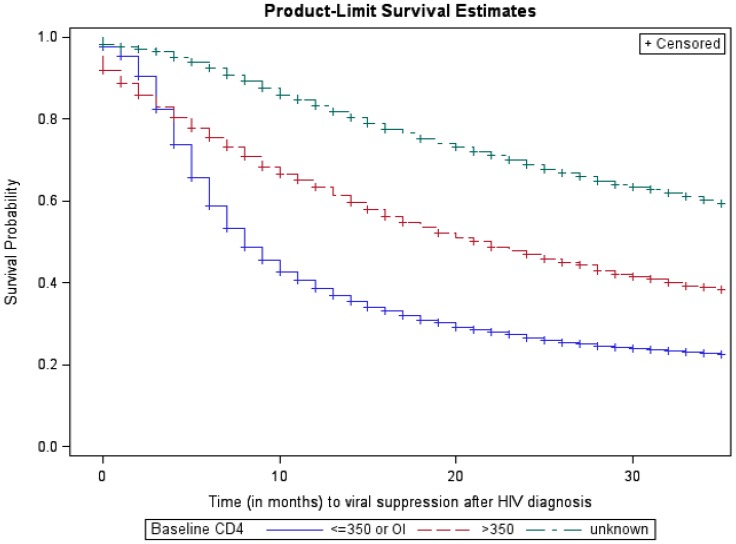
Kaplan-Meier survival curve for time from HIV diagnosis to viral suppression, by baseline CD4 count.

Overall, 85.4% of persons diagnosed with HIV in 2009 had a viral load test result within the observation period; 57.0% had a suppressed viral load (Table 1). The percentage with viral suppression during the observation period was higher among persons with a lower baseline CD4 count compared to persons with a higher CD4 or unknown CD4 count (e.g., 70.4% among persons with stage 3 disease at baseline compared with 57.6% among persons with CD4 count >350 cells/µL). The median time from diagnosis to viral suppression was 19 months (Kaplan-Meier estimate), and time to viral suppression was shorter for those with entry to care within 3 months of diagnosis compared with entry after 3 months of diagnosis (Figure 1), with a median time of 12 months for persons with entry within 3 months of diagnosis. Time to viral suppression was also shorter for persons with a lower CD4 count at baseline (Figure 2), with a median of 8 months for persons with CD4 count ≤350 cells/µL at baseline (Kaplan-Meier estimate) compared with the 19 months in the overall sample. During the first 12 months after diagnosis, persons linked to care within 3 months experienced shorter times to viral suppression (hazard ratio [HR] = 4.84 compared to those not linked to care within 3 months of diagnosis; 95% confidence interval [CI] 4.27, 5.48) (Table 2). Linkage to care within 3 months of diagnosis had an attenuated effect on the time to viral suppression in the time period 12 months after diagnosis (HR = 1.45, 95% CI 1.29, 1.62). Persons with a higher number of time-updated care visits also experienced a shorter time to viral suppression (HR = 1.51 per additional visit, 95% CI 1.49, 1.52) (Table 2).

**Table 2:** Factors associated with viral suppression^a^ among adolescents and adults diagnosed with HIV infection^b^, 2009, 19 U.S. jurisdictions.

	Adjusted Cox proportional hazard model,entry to care		Adjusted Cox proportional hazard mode,number of care visits	
Characteristic	Hazard ratio^c^ (95% CI)	P-value	Hazard ratio^c^ (95% CI)	P-value
**Sex**				
Female	1.25 (1.18 to 1.33)	<.0001	1.27 (1.20 to 1.35)	<.0001
Male	Reference		Reference	
**Age at diagnosis (per 10 years)**	1.09 (1.07 to 1.11)	<.0001	1.08 (1.06 to 1.10)	<.0001
**Race/ethnicity**				
Black/African American	0.72 (0.69 to 0.76)	<.0001	0.74 (0.70 to 0.78)	<.0001
Hispanic/Latino	0.91 (0.86 to 0.97)	0.0032	0.87 (0.82 to 0.92)	<.0001
Other	1.00 (0.91 to 1.10)	0.9495	0.94 (0.85 to 1.03)	0.1874
White	Reference		Reference	
**Transmission category**				
Injection drug use	0.78 (0.70 to 0.87)	<.0001	0.66 (0.59 to 0.74)	<.0001
Male-to-male sexual contact andinjection drug use	0.87 (0.76 to 0.99)	0.0394	0.86 (0.75 to 0.98)	0.0232
Heterosexual contact	0.87 (0.81 to 0.94)	0.0004	0.86 (0.80 to 0.92)	<.0001
Other^d^	0.80 (0.76 to 0.85)	<.0001	0.83 (0.78 to 0.87)	<.0001
Male-to-male sexual contact	Reference		Reference	
**Baseline CD4 count** e				
<200 cells/µL or Stage 3, AIDS	1.77 (1.68 to 1.86)	<.0001	1.57 (1.49 to 1.66)	<.0001
200–350 cells/µL	1.66 (1.56 to 1.76)	<.0001	1.65 (1.55 to 1.75)	<.0001
Unknown	1.10 (1.01 to 1.20)	0.0323	1.21 (1.13 to 1.29)	<.0001
>350 cells/µL	Reference		Reference	
**Care entry within 3 months of diagnosis** f				
Early time period (0–12 months)	4.84 (4.27 to 5.48)	<.0001		
Later time period (13–35 months)	1.45 (1.29 to 1.62)	<0.0001		
**The cumulative number of care** **visits after diagnosis** g			1.51 (1.49 to 1.52)	<.0001

^a^ Viral suppression defined as < = 200 copies/ml.

^b^ 17,028 adolescents and adults (aged >12 years) diagnosed in 2009, with followed up through December 31, 2011; 7, 324 censored (43.01%).

^c^ A hazard ratio >1 indicates a higher rate of viral suppression per unit time among the group of interest (e.g., those with early care entry vs. those who did not). Hazard ratio is adjusted for all other covariates in the table.

^d^ Includes 5,648 persons with unknown transmission category.

^e^ CD4 test result or opportunistic illnesses within 3 months of diagnosis; persons without a CD4 test result or OI within 3 months of diagnosis are classified as “unknown”.

^f^ Defined as ≥1 CD4 or VL test result within 3 months of diagnosis.

^g^ The number of care visits is a time-updated covariate in the model because it accumulates over time. Hazard ratio per additional visit.

Other factors associated with shorter time to viral suppression, based on the entry to care model, included sex (female versus male HR = 1.25, 95% CI 1.18, 1.33), older age (HR = 1.09 per 10 years, 95% CI 1.07, 1.11), and more advanced disease at diagnosis (lower CD4 count; for example, HR = 1.77 for persons with CD4 count <200 cells/µL or opportunistic illness versus persons with CD4 count >350 cells/µL; 95% CI 1.68, 1.86). Time to viral suppression was longer among blacks compared with whites (HR = 0.72, 95% CI 0.69, 0.76).

In a sensitivity analysis excluding the 1,885 (11.1%) of persons with suppressed viral load or censored (dead) in less than 3 months from diagnosis and using data for the remaining 15,143 persons, the HR was attenuated but provided the same conclusion for entry to care (adjusted HR = 3.47, 95% CI 3.04, 3.96 for early time period [3–12 months]). The adjusted HR was similar for the number of care visits (HR = 1.49, 95% CI 1.47, 1.50).

## Discussion

Using population-based data from the National HIV Surveillance System, study findings provide the first empirical evidence to validate that linkage to care within 3 months of diagnosis is significantly associated with more timely viral suppression, providing validation for the linkage goal of the National HIV/AIDS Strategy and the related Institute of Medicine core indicator [12], [13]. The focus for public health should be on early clinical care; however, most studies focus on viral suppression only after care entry, such as many prior publications on the continuum of care [2]–[4]. The time from diagnosis to viral suppression is a novel metric that surveillance can capture, spanning the anchoring points on both ends of the HIV care continuum. While similar findings from an earlier study were based on a clinic population that, per definition, was in care [10], these new findings support that, on a population level, prompt linkage improves outcomes with implications for population health and secondary prevention including treatment as prevention approaches. As new testing technologies can diagnose HIV at earlier stages, when persons may be more infectious and may continue to engage in risk behavior, prompt linkage and viral suppression may have an even larger impact on reducing HIV transmission in the future. While coordination of care across agencies and jurisdictions has varied historically, shortening the time of entry to care will require integration of testing, prevention and treatment services across public health departments, community-based organizations, and medical clinics.

Beyond timely linkage to care, better early retention in care, as measured by the cumulative number of care visits following diagnosis, was also associated with faster time to viral suppression. This likely reflects more expeditious initiation of ART among persons seen more often. As treatment guidelines now recommend ART initiation at any CD4 count [16], [17], our findings suggest the importance of timely care entry and early retention will become even more important going forward. While the time to viral suppression for the overall population was 19 months, among those with an initial CD4 count ≤350 cells/µL, in whom ART initiation was clearly recommended by treatment guidelines during the study period (2009), the median time to suppression was only 8 months. In the future, it is anticipated that ART will be started earlier for all newly diagnosed patients, regardless of CD4 count, such that the notable gap observed in time to viral suppression according to CD4 count is expected to diminish considerably. More timely viral suppression should become achievable for all patients, with care entry within 3 months of diagnosis and more frequent attendance at HIV care visits playing a prominent role in achieving this goal.

In addition, our analyses revealed disparities in time to viral suppression by sex, age, race/ethnicity, and transmission category, another major focus of the National HIV/AIDS Strategy. Ensuring that people receive care and treatment is critical to increase the proportion of HIV-infected individuals who achieve a suppressed viral load and to realize the benefits of treatment to reduce HIV transmission in the United States. Improvements are also needed to address disparities in care and treatment.

Overall, a high proportion of persons diagnosed with HIV are linked to care within 3 months, similar to earlier findings based on HIV surveillance and a meta-analysis of published reports [7], [18], and the majority had a suppressed viral load within the observation period. Viral suppression among the newly diagnosed persons included in the analyses was higher than the estimated percentage with viral suppression previously reported for the entire U.S. population of persons living with diagnosed HIV (30.5%–34.9% [2], [3]), but our results were similar to viral suppression reported for persons in care (62%–73% [2], [19]). This may reflect earlier findings indicating that persons who are more recently diagnosed or who have been in care for a shorter period of time are more likely to be in continuous care [6], [20]. Lack of viral suppression or difference in time to viral suppression among subgroups may be due to delayed prescription or adherence to ART or barriers to accessing care. A limitation of surveillance data is the lack of information regarding ART utilization and adherence, such that the role of these steps on the HIV care continuum could not be evaluated. However, surveillance is unique in the capture of both dates of HIV diagnosis as well as viral load measures allowing measurement of the time interval between these two points on either end of the continuum. We suggest this time interval may serve as a novel, valuable indicator for longitudinal monitoring using population-based HIV surveillance data at a jurisdiction, state and national level.

Study results reflect the prioritization of treatment for persons with more advanced disease, with shorter times to viral suppression for those with advanced disease compared with persons with CD4 counts >350 cells/µL at diagnosis. Indeed, in sensitivity analyses restricted to participants with initial CD4 counts ≤350 cells/µL the median time to viral suppression was less than half the time observed for the overall sample (8 vs. 19 months by Kaplan-Meier estimate). Treatment guidelines at the time recommended ART prescription based on stage of disease and other relevant factors [21], [22]. Because guidelines now recommend universal offering of treatment, it is anticipated that timely care entry and regular visit frequency will become even more paramount to ART uptake and success [16], [17].

Our results are similar to earlier findings, conducted among persons who had entered HIV medical care, among whom a lower percentage of viral suppression was observed in blacks compared with whites and among persons with HIV infection attributed to transmission other than male-to-male sexual contact [2], [7]. Factors that may contribute to reduced access to care and treatment include lack of social support, competing child care responsibilities, food insecurity, unstable housing, lack of transportation, lower education, poverty, unemployment, homelessness, and mental health or substance abuse problems [23], [24]. In addition, differences exist in health insurance coverage, with 32% of Latinos, 21% of blacks, and 16% of whites being uninsured [25]. Clearly, improvements are needed to achieve national goals of alleviating disparities in HIV care and outcomes, with these population-based data from newly diagnosed persons corroborating clinic-based data among those in care.

Our analysis was subject to several limitations. Data were available from a limited number of areas; the 19 jurisdictions represent 37.5% of all diagnoses in 2009 in the United States. However, the demographic characteristics were similar among the persons included in the analyses compared to the overall population diagnosed with HIV in the United States in 2009. As additional areas improve laboratory reporting to CDC, future estimates of care utilization will be more representative. Information was also not available to determine whether CD4 or viral load tests were ordered by emergency departments or inpatient settings, and people with HIV may have CD4 or viral load tests performed but fail to attend the outpatient visit. However, surveillance data provide information on care measures that are not subject to limitations of patient recall or appointment tracking and measures can be consistently applied to all locations. Numbers were too small to report HRs for other individual race groups. For persons who move to another jurisdiction after HIV diagnosis, surveillance protocols call for reporting of information from both jurisdictions including, at minimum, the first CD4 count (any value and the first CD4 count <200 cells/µL at any time) and viral load test results after first diagnosis in the jurisdiction. Persons are de-duplicated in the national surveillance database while maintaining information reported from all jurisdictions. The percentage of persons who moved during the follow-up time is expected to be small; however, for persons with unsuppressed viral load who moved to another jurisdiction where viral suppression was achieved it is possible that information was not reported to CDC. This could result in an underestimation of the effect if care was accessed early.

In summary, the results support strengthening efforts to improve engagement in care among persons newly diagnosed with HIV, validating the importance of the Institute of Medicine core indicator of linkage to care within 3 months. Effective interventions need to assure not only prompt linkage but also that subsequent retention in care is established after the first visit [26]. A review of interventions has shown that strategies exist that can improve retention in care [27]. Such strategies include case management building on patients’ strengths, peer navigation, reducing structural- and system-level barriers, and effective communication between patients and providers [27]–[32]. Successful implementation of such strategies is imperative to achieving the goals of the National HIV/AIDS Strategy, and to maximizing domestic HIV treatment and prevention success.
